# Cerebral Hemodynamic and Neurotrophic Factor Responses Are Dependent on the Type of Exercise

**DOI:** 10.3389/fphys.2020.609935

**Published:** 2021-01-21

**Authors:** Samuel R. Weaver, Bethany D. Skinner, Rhodri Furlong, Rebekah A. I. Lucas, N. Timothy Cable, Catarina Rendeiro, Helen M. McGettrick, Samuel J. E. Lucas

**Affiliations:** ^1^School of Sport, Exercise and Rehabilitation Sciences, College of Life and Environmental Sciences, University of Birmingham, Birmingham, United Kingdom; ^2^Centre for Human Brain Health, University of Birmingham, Birmingham, United Kingdom; ^3^College of Medical and Dental Sciences, Institute of Inflammation and Ageing, University of Birmingham, Birmingham, United Kingdom

**Keywords:** cerebrovascular, exercise, hemodynamic, neurotrophic factor, physiology

## Abstract

This study examined acute cerebral hemodynamic and circulating neurotrophic factor responses to moderate intensity continuous exercise (MICT), guideline-based high intensity interval exercise (HIIT), and sprint interval exercise (SIT). We hypothesized that the pattern of middle cerebral artery velocity (MCAv) response would differ between interval and continuous exercise, with SIT inducing the smallest increase from rest, while increases in neurotrophic factors would be intensity-dependent. In a randomized crossover design, 24 healthy adults (nine females) performed three exercise protocols: (i) MICT (30 min), (ii) HIIT (4 × 4 min at 85% HR_max_), and (iii) SIT (4 × 30 s supramaximal). MCAv significantly increased from rest across MICT (Δ13.1 ± 8.5 cm⋅s^–1^, *p* < 0.001) and all bouts of HIIT (Δ15.2 ± 9.8 cm⋅s^–1^, *p* < 0.001), but only for the initial bout of SIT (Δ17.3 ± 11.6 cm⋅s^–1^, *p* < 0.001). Immediately following each interval bout, MCAv increased (i.e., rebounded) for the SIT (9–14% above rest, *p* ≤ 0.04), but not HIIT protocol. SIT alone induced significant elevations from rest to end-exercise in vascular endothelial growth factor (VEGF; Δ28 ± 36%, *p* = 0.017) and brain-derived neurotrophic factor (BDNF, Δ149% ± 162%, *p* < 0.001) and there were greater increases in lactate than in either other protocol (>5-fold greater in SIT, *p* < 0.001), alongside a small significant reduction at the end of active recovery in insulin-like growth factor 1 (IGF-1, Δ22 ± 21%, *p* = 0.002). In conclusion, while the nature of the response may differ, both guideline-based and sprint-based interval exercise have the potential to induce significant changes in factors linked to improved cerebrovascular and brain health.

## Introduction

Physical activity is unique in its ability to positively impact human health and promote wellbeing across a lifespan (Pedersen and Saltin, 2015; O’Donovan et al., 2017; Kraus et al., 2019). These benefits are clearly seen within the brain, with higher physical fitness positively correlated with brain health and a reduction in risk and progression rate of a number of neurological diseases (Kramer et al., 2006; Bailey et al., 2013; Burley et al., 2016).

Traditional guidelines, typified by moderate intensity, and continuous efforts of 30 min, elicit beneficial changes in resting cerebral blood flow, cognitive function, cardiovascular health, and all-cause mortality (Garber et al., 2011; Cai et al., 2017; Alfini et al., 2019). However, poor adherence to traditional exercise strategies, often characterized by a lack of time (Costello et al., 2011), has resulted in increased interest in high intensity, interval-based strategies, which have shown similar health and fitness benefits compared to moderate intensity steady-state exercise (Stork et al., 2017; Gibala and Little, 2020). Despite this, studies focusing on the cerebrovascular responses to these strategies are limited in number. While current evidence indicates comparable middle cerebral artery velocity (MCAv) responses between continuous and interval-based moderate intensity exercise (Klein et al., 2019), MCAv has been shown to be suppressed over the course of a single 30 s all-out sprint bout, followed by a marked elevation in MCAv during recovery (Curtelin et al., 2018; Labrecque et al., 2020). This reduction in response to high intensity interval-based strategies is in contrast to responses within the peripheral vasculature (Ramos et al., 2015), likely driven by the unique response of the cerebrovasculature to exercise-induced hyperventilatory hypocapnia (Ogoh and Ainslie, 2010), as well as differences in the regulatory responses to elevated systemic flow and blood pressure (Brassard et al., 2017; Ogoh et al., 2018; Calverley et al., 2020). Regardless of the mechanisms driving these differences, it is essential that we understand how choice of exercise strategy can alter hemodynamic responses, as acute elevations in blood flow are likely pivotal in determining the adaptive benefits of longer training periods (Green et al., 2017). Understanding this relationship is particularly important as research increasingly supports short (30–60 s) sprint interval training (SIT) protocols at supramaximal intensities, rather than longer (∼4 min) high intensity efforts performed in guideline-based high intensity interval training (HIIT) (Weston et al., 2014). While the current literature demonstrates the complex relationship between SIT and measures of cerebral hemodynamics (Curtelin et al., 2018; Labrecque et al., 2020), it is not clear whether these responses change over multiple bouts as is typical in training interventions, nor how SIT- and HIIT-based strategies may differ in their response.

Exercise also has the capacity to alter the profile of circulating signaling factors, including neurotrophic factors with the potential to act within the brain (Voss et al., 2013b). Amongst these, brain-derived neurotrophic factor (BDNF), insulin-like growth factor 1 (IGF-1), and vascular endothelial growth factor (VEGF) are particularly relevant due to their ability to elicit a comprehensive profile of responses within the cerebrovasculature, blood-brain barrier, and in brain plasticity (Voss et al., 2013b; Basso and Suzuki, 2017). Increases in all three of these factors have been demonstrated under a single exercise bout, with responses appearing to be intensity-dependent (Wahl et al., 2010, 2014; Jeon and Ha, 2017; Reycraft et al., 2020).

While the origin and brain-specific changes in many of these factors is not fully understood, particularly in relation to BDNF (Rasmussen et al., 2009; Klein et al., 2011), the impact that exercise can have on these factors is increasingly clear. In both animal models and humans, increases in BDNF have been associated with improvements in brain function, acute post-exercise cognitive function, and subsequent adaptive benefits within the brain (Voss et al., 2013a; Basso and Suzuki, 2017; Kujach et al., 2020). Similarly, although acute changes in IGF-1 do not appear to correlate with changes in cognition (Tsai et al., 2014), more chronically it has been suggested that IGF-1 is central to healthy cognitive aging, due to its role in regulating metabolism and insulin resistance in the brain (Lewitt and Boyd, 2019). Further, IGF-1 signaling is essential to the maturation of BDNF and so may play a secondary role in stimulating its release (Carro et al., 2000). VEGF is most widely studied in terms of vascular endothelial regulation, stimulating angiogenic signal pathways and vascular adaptation to alterations in flow (Olsson et al., 2006). A number of animal studies have also demonstrated its role within the brain, promoting angiogenesis within the cerebrovasculature (Morland et al., 2017; Geiseler and Morland, 2018) and exhibiting neuroprotective effects when upregulated (Jin et al., 2002; Góra-Kupilas and Jośko, 2005).

The evolving role of lactate as a signaling molecule raises interesting prospects within the brain, particularly considering its exponential increases seen at higher intensities (Hughson et al., 1987). Increases in lactate alone have the capacity to significantly increase BDNF (Schiffer et al., 2011) and have been associated with exercise-induced changes in circulating BDNF, IGF-1, and VEGF (Kujach et al., 2020). Furthermore, lactate has been shown to modulate brain signaling pathways, crossing the blood-brain barrier and binding to HCAR1 receptors that are highly expressed within the brain (Lauritzen et al., 2014), driving responses relating to cerebral angiogenesis and stimulating cerebral VEGF expression (Morland et al., 2017).

Given the potential impact of exercise intensity and protocol on cerebrovascular function, this study aimed to examine the MCAv response to moderate intensity continuous exercise (MICT), guideline-based high intensity interval exercise (HIIT), and sprint interval exercise (SIT). Secondly, we aimed to explore how these exercise protocols may differentially alter the acute profile of key neurotrophic markers, which have previously been linked to exercise-induced changes in brain health. It was hypothesized that the pattern of middle cerebral artery velocity (MCAv) response would differ between interval and continuous exercise, with SIT inducing the smallest increase from rest. In addition, we hypothesized that increases in circulating neurotrophic factors would be intensity-dependent, with SIT inducing the greatest response.

## Materials and Methods

### Participants

Twenty-four regularly active participants were recruited for this study (see Table 1 for participant characteristics). All participants were healthy, with no history of cardiovascular, cerebrovascular, or respiratory disease, and were not taking any medication (with the exception of oral contraception in female participants). Visits were carried out within an hour of the same time of day for each participant, with a minimum of 48 h between sessions. The study was approved by the University of Birmingham Ethics Committee (ERN-17_1570) and all participants gave written, informed consent prior to enrolling in the study, in adherence with the Declaration of Helsinki.

**TABLE 1:** Baseline characteristics and resting values for participants taking part in three different exercise protocols.

Baseline characteristics (*n* = 24; 9 female)	Mean	*SD*
Age (years)	23	5
Body Mass (kg)	73.0	11.1
VO_2peak_ (mL⋅min^–1^)	3,185	544
VO_2peak_ (mL⋅kg^–1^⋅min^–1^)	43.9	6.4

**Resting values**		

**MICT**		
MCAv (cm⋅s^–1^)	72.0	13.6
P_ET_CO_2_ (mmHg)	33.8	3.6
HR (beats⋅min^–1^)	68	10
BDNF (pg⋅mL^–1^)	112.5	75.8
IGF-1 (ng⋅mL^–1^)	1,960	1,350
VEGF (pg⋅mL^–1^)	456.5	980.6
Lactate (mM)	1.1	0.2
**HIIT**		
MCAv (cm⋅s^–1^)	70.4	11.4
P_ET_CO_2_ (mmHg)	33.6	3.6
HR (beats⋅min^–1^)	67	12
BDNF (pg⋅mL^–1^)	134.0	91.6
IGF-1 (ng⋅mL^–1^)	1,905	1,160
VEGF (pg⋅mL^–1^)	404.9	770.8
Lactate (mM)	1.1	0.3
**SIT**		
MCAv (cm⋅s^–1^)	70.5	11.8
P_ET_CO_2_ (mmHg)	33.4	3.2
HR (beats⋅min^–1^)	71	10
BDNF (pg⋅mL^–1^)	134.8	104.5
IGF-1 (ng⋅imL^–1^)	2,101	1,444
VEGF (pg⋅mL^–1^)	369.2	740.1
Lactate (mM)	1.3	0.9

*SD, standard deviation; MICT, moderate intensity continuous training; HIIT, high intensity interval training; SIT, sprint interval training; MCAv, middle cerebral artery velocity; P_*ET*_CO_2_, partial pressure of end-tidal carbon dioxide; HR, heart rate; BDNF, brain-derived neurotrophic factor; IGF-1, insulin-like growth factor 1; VEGF, vascular endothelial growth factor.*

### Experimental Protocol

**FIGURE 1:**
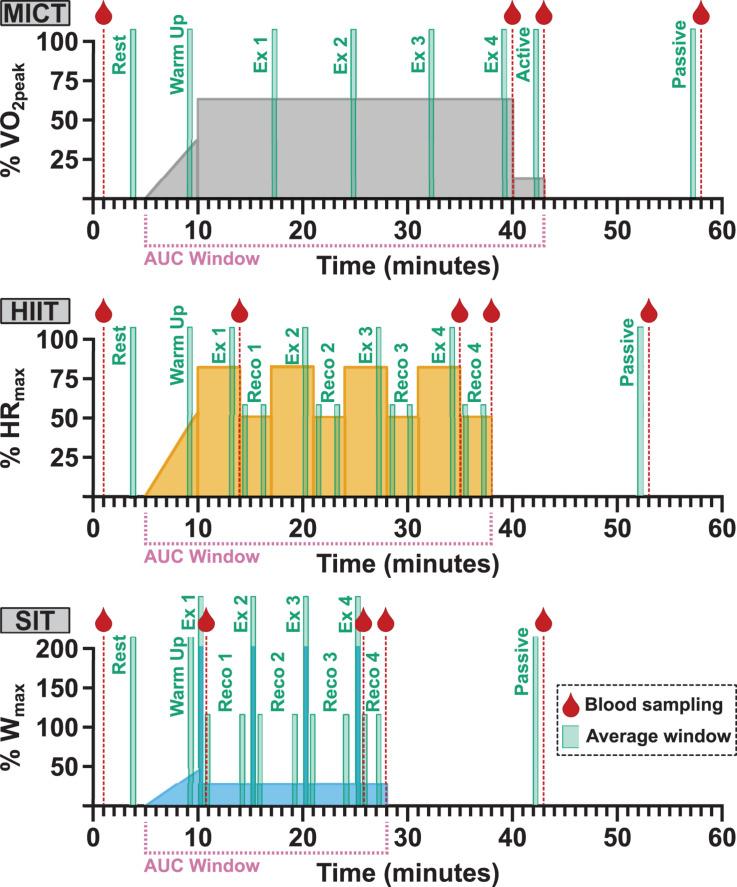
Participants took part in three separate exercise visits in a randomized order, consisting of 30 min of moderate, steady-state exercise at 65% VO_2peak_ (MICT); four 4 min high intensity intervals at 85% HR_max_ (HIIT); and four 30 s supramaximal sprint intervals at 200% W_max_ (SIT). All protocols were preceded by 5 min of seated rest and a 5 min warm-up, and followed by 3 min of active recovery at 50 W and 15 min of seated passive recovery. The timing of blood sampling (red droplet), data averaging windows, and labels for analysis (green boxes) and the period used in area under the curve (AUC) analysis (pink bracket) are included for all protocols. Averaging was carried out over 30-second windows across the course of each exercise protocol (Ex) and during the initial and final minute of recovery (Reco) following each HIIT and SIT bout. The final 30-second recovery window in both HIIT and SIT was taken as the active recovery timepoint, in conjunction with the active recovery window (Active) in MICT. VO_2peak_, maximum oxygen consumption; HR_max_, maximum heart rate; W_max_, maximum aerobic power.

Following enrolment, participants attended the laboratory on four separate occasions to carry out: (1) a ramp-increment aerobic capacity test (VO_2peak_ test); (2) 30 min of moderate-intensity steady-state exercise (MICT); (3) four 4 min high intensity interval (HIIT) workouts based on a clinical intervention model (Weston et al., 2014); and (4) four 30 s all-out sprints (SIT) (Figure 1). Participants were asked to refrain from vigorous exercise or consuming alcohol for 24 h and from consuming caffeine within 12 h of each visit. In addition, participants were asked to record any food consumed on each visit day and to refrain from consuming food within 2 h of testing beginning, with the same foods being consumed prior to subsequent sessions.

### Maximal Oxygen Consumption Test (VO_2peak_)

VO_2peak_ was assessed using a ramp-incremental exercise protocol on a cycle ergometer (Excalibur Sport, Lode, Netherlands). This test consisted of a 5 min warm up until the participant indicated a rate of perceived exertion (RPE) of 11 on a Borg Visual Scale (Borg, 1982), followed by a 3 min ramp protocol in which resistance was increased by 30 W every 3 min until volitional fatigue was reached, or the participant was not able to maintain a consistent cadence above 50 RPM. Oxygen consumption was continuously measured (Vyntus CPX, Vyaire Medical, United Kingdom) and VO_2peak_ was defined as the highest 30 s average achieved during the test. Continuous measures of HR (Polar H9 HR Sensor, Polar, Finland) were collected for determination of HR_max_. Maximal power output W_max_ was determined as the power output achieved during the final stage of the VO_2peak_ test.

### Experimental Visits

All three experimental visits were carried out in a randomized, cross-over fashion, with the order of experimental visits for all participants determined *a priori* using customized R script (R Core Team, 2019). On arrival, an intravenous catheter (20G, BD Venflon, United Kingdom) was placed in the antecubital vein of the forearm to enable blood sample collection during exercise; participants then rested in a supine position for a minimum of 10 min before resting blood samples were collected. Participants were instrumented for cerebrovascular and cardiorespiratory measures (detailed below) and sat quietly for 5 min while resting measures of middle cerebral artery velocity (MCAv), heart rate (HR), and end-tidal carbon dioxide (P_ET_CO_2_) were collected. All exercise protocols were carried out at a self-selected cadence, included a 5 min warm up identical to that used during VO_2peak_ testing, and ended with 3 min of active recovery at 50 W and a seated 15 min passive recovery period (Figure 1). Verbal encouragement was given throughout all exercise protocols and participants were given information about elapsed time at regular intervals.

#### MICT Protocol

During MICT, participants carried out 30 min of continuous exercise at 65% of VO_2peak_. Resistance on the cycle ergometer was initially set between 55 and 60% of W_max_ and adjusted once cycling had commenced in order to attain 65% of VO_2peak_. Oxygen consumption was continuously monitored across the duration of the 30 min period to ensure this intensity was maintained and cycling resistance was adjusted as required.

#### HIIT Protocol

HIIT consisted of four 4 min intervals at 85% HR_max_, separated by 3 min of active recovery at 50 W, based on guideline recommendations and definitions of HIIT in clinical settings (Weston et al., 2014). Initial workload during interval bout 1 was set at ∼70% W_max_, and monitored and adjusted to achieve 85% HR_max_ in a similar manner to the MICT workload. For remaining bouts, the initial intensity was set at the power output during the final 30 s of the preceding 4 min bout and adjusted as necessary to maintain intensity at 85% HR_max_.

#### SIT Protocol

SIT intervals consisted of four 30 s intervals at 200% of W_max_, separated by 4.5 min of active recovery at 50 W, with the exception of the final recovery which was matched to MICT and HIIT at 3 min at 50 W. Following warm-up, the workload was reduced to 50 W for 30 s in order to ensure that the change in workload from pre-interval to sprint was matched across all four intervals. Workload was ramped up 5 s prior to each sprint interval in order to account for a ∼5–7 s delay in resistance on the cycle ergometer and intensity was dropped to 50 W as soon as the 30 s bout ended.

### Measurements

#### Cerebral Blood Velocity

MCAv was assessed bilaterally using a 2 MHz transcranial doppler (TCD) ultrasound system (Multi Dop X, DWL, Compumedics Ltd., Germany). Ultrasound probes were attached to a fully adjustable headset (DiaMon, DWL) to allow for continuous measurement during exercise. The MCA was located via the temporal window using standardized search techniques, with depth, gain, and filter settings matched within participants across visits (Willie et al., 2011). Resting values for right and left MCAv were compared during rest to maximize comparability between sessions, with the most consistent MCA (right/left) signal across all three sessions used for data analysis.

#### Respiratory Gases and Ventilation Rate

Breath-by-breath measures of respiratory gases were measured by collection of expired air through a facemask (Hans Rudolph, United States) and analyzed continuously via a metabolic cart (Vyntus CPX, Vyaire Medical). Measures of minute ventilation (V_E_), oxygen consumption (VO_2_), and P_ET_CO_2_ were exported for later analysis.

#### Data Acquisition

MCAv and HR data were recorded through an analog-to-digital converter (Powerlab, ADInstruments, New Zealand) and stored for further analysis using dedicated signal analysis software (LabChart Pro v7, ADInstruments). P_ET_CO_2_ data were aligned with other signal data and processed alongside MCAv to match time course and data averaging periods between the two.

#### Blood Sampling and Analysis

Blood samples were drawn from the antecubital vein and collected into two 6 mL vacutainers for the isolation of plasma, one containing K_2_EDTA and one lithium heparin (LH) as an anticoagulant (both BD Vacutainer, United Kingdom). Samples were collected at rest, at the end of each exercise protocol, end of active recovery, end of passive recovery, and in the case of HIIT and SIT an additional aliquot was taken following the initial interval bout (Figure 1). Samples were immediately centrifuged at 5,000 g, for 10 min at 4°C, carefully aliquoted to avoid disruption of buffy coat, and frozen at −80°C for future analysis. All analyses were conducted using plasma isolated with LH as an anticoagulant, with the exception of VEGF, which was analyzed using plasma isolated in K_2_EDTA.

Plasma levels of VEGF, IGF-1, and BDNF were analyzed by DuoSet ELISA according to manufacturer’s instructions (#DY293B, #DY291, #DY248, respectively, all R&D Systems, United Kingdom), with each sample run in duplicate on a single plate. Sample concentration was determined using standard curves produced by 4-parameter logistic curve-fitting and automated concentration analysis (AssayFit Pro, Assay Cloud, Netherlands). In the eventuality that all samples from a single visit showed no detectable protein-of-interest, samples were re-run, using a new sample aliquot on a second plate alongside samples that reported reproducible levels of the given protein-of-interest. All sample aliquots used within these analyses were subject to a single freeze-thaw cycle.

Circulating levels of blood lactate were analyzed using an automated photometric-based chemical analyzer (RX+ Daytone, Randox, United Kingdom), using purpose made assay kits, standards, and reagents, according to the manufacturer’s guidelines (Randox).

### Data Processing and Statistical Analysis

#### Data Processing

Power output data was collected across all three experimental visits and averaged over 5 min periods across the duration of MICT and over the duration of each bout of HIIT and SIT. Additionally, average power output was calculated over the complete duration of each protocol.

Hemodynamic and respiratory data were averaged over the penultimate 30 s of rest, warm-up, and active and passive recovery. During the exercise efforts, data were averaged over 30 s at four time points (7.5 min intervals) during MICT, over the penultimate 30 s of HIIT bouts, and across the full 30 s duration of SIT bouts. In addition, 30 s averages were obtained in the initial and final minute of recovery following each HIIT and SIT bout (RECO 1–4; see Figure 1).

MCAv data were also exported at 1 Hz continuously from the start of warm-up to the end of active recovery to plot time-course data for the duration of each study visit, alongside quantification of area-under-curve (AUC) for the same time-course, using Pulse Analysis software within Labchart (ADInstruments). AUC was calculated as total blood velocity (cm⋅s^–1^) over the total time period (s) and so is presented as a measure of displacement (cm⋅s^–1^ ⋅ s = cm).

#### Statistical Analysis

Assumptions of normality were assessed by graphical interpretation of density distribution and Q-Q plots, as well as by analysis of skewness, kurtosis, and Shapiro-Wilk’s Test statistics. Resting baseline data and power output data were compared by one-way analysis of variance (ANOVA); if sphericity assumptions were not met under Mauchly’s Test then Greenhouse-Geisser corrections were utilized for ANOVA results.

Comparison of repeated-measures outcome data was carried out using linear mixed effects models (LMEM), with time and protocol included as fixed factors and participant ID as a random factor. Inclusion of sex and fitness (VO_2peak_) as factors was assessed using Akaike’s Information Criteria (AIC) to determine inclusion and weighting of model errors for each outcome variable (Symonds and Moussalli, 2011). *Post hoc* testing for interactions within ANOVA and LMEM were carried out with pairwise comparison and Tukey Test adjustments for multiple comparison. All results are presented as mean ± standard deviation (mean ± *SD*) unless otherwise stated.

## Results

### Exercise Intensity During Acute Exercise

Mean power output across the duration of MICT was 148 ± 28 W and did not differ significantly across the duration of the protocol (*p* > 0.05). During HIIT, average power output was 198 ± 45 W and remained consistent across bouts 1–3 (201 ± 44 W; 201 ± 46 W; 198 ± 46 W, respectively), although a significant decline was seen in bout 4 compared to the other 3 bouts (194 ± 47 W, *p* ≤ 0.02). Due to the design of the SIT condition, average power output was matched across all four bouts (503 ± 103 W). Average power output was significantly lower in MICT than in either other condition (*p* < 0.001), and significantly greater in SIT than in HIIT (*p* < 0.001).

Oxygen consumption was significantly elevated from resting baseline across all three protocols (*p* < 0.001, Table 2). During MICT, V̇O_2_ remained relatively stable across the course of 30 min, although there was a small significant increase in the third time point (2,063 ± 367 mL⋅min^–1^, *p* ≤ 0.03) compared with time points one and two (1,975 ± 354 mL⋅min^–1^ and 2,006 ± 384 mL⋅min^–1^, respectively). Oxygen consumption during both HIIT and SIT was significantly greater than MICT at all exercising time points (*p* ≤ 0.046, Table 2). During HIIT, V̇O_2_ was significantly lower in bout 1 (2,327 ± 574 mL⋅min^–1^) than either bout 2 (2,447 ± 511 mL⋅min^–1^, *p* = 0.04) or bout 3 (2,483 ± 511 mL⋅min^–1^, *p* = 0.007). Similarly, SIT bout 1 (2,291 ± 392 mL⋅min^–1^) was significantly lower than bout 2 (2,512 ± 470 mL⋅min^–1^, *p* ≤ 0.001) and bout 4 (2,473 ± 502 mL⋅min^–1^, *p* < 0.03).

**TABLE 2:** Measures of oxygen consumption, minute ventilation, and heart rate at rest and across the duration of three different exercise protocols.

	Rest	Ex 1	Ex 2	Ex 3	Ex 4
**MICT**					
VO_2_ (mL⋅min^–1^)	293 ± 112	1,975 ± 354*^$†^	2,006 ± 384*^$†^	2,063 ± 367*^$†^	2,023 ± 383*^$†^
V_E_ (L⋅min^–1^)	9.4 ± 3.1	55.1 ± 10.9*^$†^	56.1 ± 12.2*^$†^	57.7 ± 11.9*^$†^	57.4 ± 13.6*^$†^
HR (beat⋅min^–1^)	68 ± 10	144 ± 14*^$†^	147 ± 13*^$†^	150 ± 13*^$†^	151 ± 14*^$†^
**HIIT**					
VO_2_ (mL⋅min^–1^)	287 ± 69	2,327 ± 574*	2,447 ± 511*	2,484 ± 511*	2,396 ± 631*
V_E_ (L⋅min^–1^)	9.5 ± 2.1	70.0 ± 21.2*	76.9 ± 20.1*^Ω^	78.8 ± 21.3*^Ω^	77.9 ± 22.9*^Ω^
HR (beat⋅min^–1^)	67 ± 12	159 ± 10*	167 ± 10*	170 ± 12*	172 ± 11*
**SIT**					
VO_2_ (mL⋅min^–1^)	316 ± 69	2,291 ± 392*	2,512 ± 470*	2,448 ± 529*	2,473 ± 502*
V_E_ (L⋅min^–1^)	10.7 ± 2.4	83.3 ± 20.3*	101.3 ± 27.6*	106.0 ± 26.4*	109.8 ± 28.8*
HR (beat⋅min^–1^)	71 ± 10	164 ± 14*	169 ± 15*	171 ± 13*	171 ± 11*

SD, standard deviation; MICT, moderate intensity continuous training; HIIT, high intensity interval training; SIT, sprint interval training; VO_2_, oxygen consumption; V_*E*_, minute ventilation; HR, heart rate. Significance (analyzed by linear mixed model, p < 0.05) between resting values and subsequent time points is denoted by *. Significant differences between protocols are denoted by ^$^*between MICT and HIIT;*^†^*between MICT and SIT; and*^Ω^*between HIIT and SIT.*

### Hemodynamics Responses to Acute Exercise Interventions

Baseline characteristics and resting values for MCAv, P_ET_CO_2,_ and HR during seated rest are displayed in Table 1. No significant differences were seen between any of these measures at rest (*p* > 0.05). Within-participant variability (coefficient of variability) among intervention visits was 5.5% for MCAv, 4.8% for P_ET_CO_2_, and 7.2% for HR.

Assessment of model fit validated the inclusion of sex in linear modeling of MCAv, P_ET_CO_2_, and HR. While significant interactions were seen between time and protocol across all outcome measures and data sets, inclusion of sex as a fixed factor did not reveal any significant main effect or interaction between sex and the time-by-protocol interaction effect. For this reason, all further analysis and interpretation of these data were carried out including male and female participants as a complete cohort.

**FIGURE 2:**
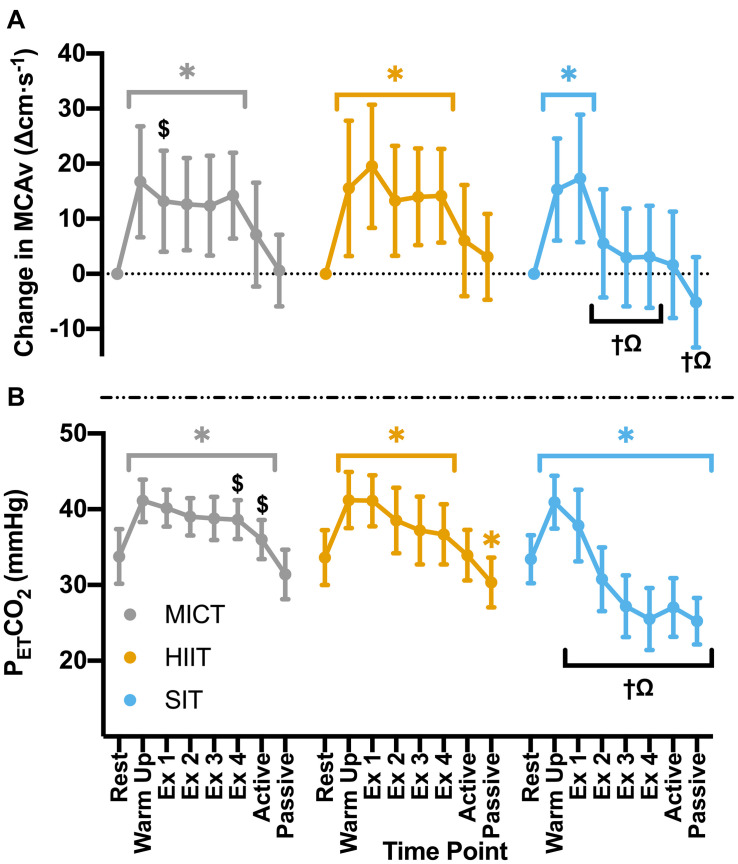
**(A)** MCAv was determined continuously by transcranial doppler ultrasound (TCD) and presented as change in MCAv from rest in each protocol for each participant. **(B)** P_ET_CO_2_ was determined through breath-by-breath respiratory gas exchange and ventilation. Data were averaged over 30 s of rest (rest); 5 min warm up (warm up); 3 min active recovery (active); and 15 min seated passive recovery (passive) in all protocols. Exercising averages (Ex 1–4) were taken every 7 min during 30 min of moderate intensity (65% VO_2peak_) steady state exercise (MICT); in the final minute of the four 4 min high intensity (85% HR_max_) interval bouts (HIIT) and across the duration of four 30 s supramaximal (200% W_max_) sprint intervals (SIT). Data are presented as mean ± *SD* (*n* = 24; except SIT–Passive: *n* = 23) and were analyzed by linear mixed models. Significant differences (analyzed by linear mixed model, *p* < 0.05) between resting values and subsequent time points are denoted by *. Significant differences between protocols are denoted by ^$^ between MICT and HIIT; ^†^ between MICT and SIT; and ^Ω^ between HIIT and SIT.

Significant increases in MCAv from rest during warm up (*p* < 0.001) were comparable across all exercise protocols (MICT: Δ16.7 ± 10.1 cm⋅s^–1^, HIIT: Δ15.5 ± 12.3 cm⋅s^–1^, SIT: Δ15.3 ± 9.3 cm⋅s^–1^, *p* > 0.05 between protocols). During MICT a sustained and uniform elevation in MCAv was seen across all time points (mean across time points: 85.1 ± 16.2 cm⋅s^–1^), with no significant changes across the 30 min time course (Figure 2). While all HIIT bouts showed a sustained, significant elevation in MCAv from rest (19–21%, *p* < 0.001), bout 1 showed significantly greater elevation in comparison to both HIIT bout 2 (Δ6.3 ± 6.4 cm⋅s^–1^, *p* = 0.043) and time point one during MICT (Δ4.8 ± 9.0 cm⋅s^–1^, *p* = 0.047). End-tidal CO_2_ showed similar patterns in response across both MICT and HIIT, with significant elevations from baseline that were sustained across warm up and all exercising time points in both protocols (Figure 2). HIIT bout 1 also showed significantly greater increases in P_ET_CO_2_ than all other HIIT bouts (≥2.6 mmHg, *p* < 0.001), whilst a small significant difference was seen between bouts 3 and 4 and the comparable timepoints during MICT (Δ1.4 ± 2.8 mmHg and Δ1.8 ± 2.9 mmHg respectively, *p* ≤ 0.04).

**FIGURE 3:**
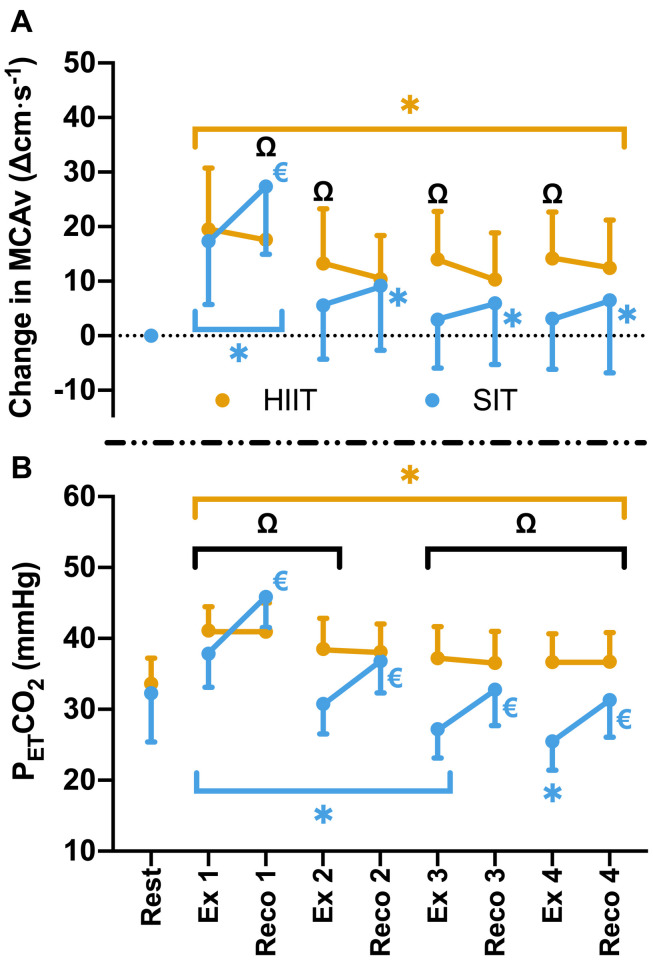
**(A)** MCAv was determined continuously by transcranial doppler ultrasound (TCD) and presented as change in MCAv from rest in each protocol for each participant. **(B)** P_ET_CO_2_ was determined by measurement of breath-by-breath respiratory gas exchange and ventilation. Data were averaged for 1 min during seated rest (Rest); over 30 s during the final minute of four 4 min high intensity (85% HR_max_) interval bouts (HIIT; Ex 1 – 4); across the duration of four 30 s supramaximal (200% W_max_) sprint intervals (SIT; Ex 1 – 4), and over a 30 s period, 15 s into the recovery following each bout in both interval protocols (Reco 1 – 4). Data are presented as mean ± SD (*n* = 24). Significance (analyzed by linear mixed model, *p* < 0.05) between time points is denoted by * for differences from resting values; ^**€**^ for differences between interval (ex) and recovery (reco). Significant differences between protocols are denoted by ^Ω^.

Compared to resting values, there was a significant elevation in MCAv following the first 30 s SIT bout (Δ17.3 ± 11.6 cm⋅s^–1^, *p* < 0.001), accompanied by a significant increase in P_ET_CO_2_ at the same time point (Δ4.5 ± 4.8 mmHg, *p* < 0.001). These changes were comparable to those seen during MICT and HIIT in terms of MCAv (both *p* > 0.05), however, P_ET_CO_2_ was significantly lower in SIT than in both MICT (Δ2.3 ± 3.9 mmHg, *p* = 0.004) and HIIT (Δ3.3 ± 3.0 mmHg, *p* < 0.001). During subsequent SIT bouts, significant reductions in MCAv were seen in comparison to bout 1 (≥11.7 cm⋅s^–1^, *p* < 0.001; Figure 2), with no further elevations above baseline across bouts 2–4 (*p* > 0.05). Significant reductions in P_ET_CO_2_ were also seen with repeated bouts, resulting in a significant suppression in P_ET_CO_2_ below resting baseline from bout 2 onwards (*p* ≤ 0.004; Figure 2). During recovery from sprint intervals, a pattern of elevated MCAv and P_ET_CO_2_ was seen within the first minute of recovery following each SIT bout, which was not seen in the same window of time during HIIT (Figure 3). This “rebound” was only found to be significant in comparison to the related sprint bout following bout 1 (Bout vs. Recovery = Δ10.0 ± 7.9 cm⋅s^–1^, *p* < 0.001), although smaller significant elevations above baseline were seen across all subsequent rebounds (9–14% above rest, *p* ≤ 0.04). All four bouts showed significant elevations in P_ET_CO_2_ during the rebound phase both in comparison to the preceding sprint bout and to baseline measures (≥5.6 mmHg, *p* < 0.001).

Analysis of V_E_ measures showed an intensity-dependent pattern across all three protocols, mirroring changes seen in P_ET_CO_2_. Specifically, significant elevations were seen in all three protocols (*p* ≤ 0.001, Table 2), but with lower elevations in MICT than either other protocol at all time points (*p* ≤ 0.01), and significantly greater V_E_ in SIT than in HIIT from bout 2 onwards (*p* ≤ 0.002). While V_E_ remained relatively stable across MICT, significant differences were seen between bout 1 of HIIT (70.0 ± 21.2 L⋅min^–1^) and all subsequent HIIT bouts (minimum 76.9 ± 20.1 L⋅min^–1^, *p* ≤ 0.001). Similarly, bout 1 of SIT (83.3 ± 20.2 L⋅min^–1^) was lower than all subsequent bouts (minimum 101.3 ± 27.6 L⋅min^–1^, *p* ≤ 0.001), and bout 2 was lower than 4 (101.3 ± 27.6 and 109.8 ± 28.7 L⋅min^–1^; respectively, *p* = 0.03).

**FIGURE 4:**
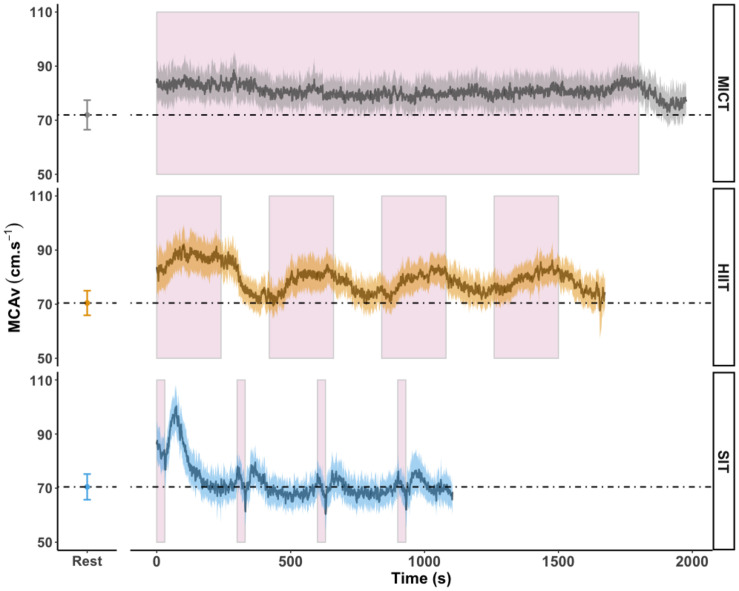
MCAv was determined continuously by transcranial doppler ultrasound (TCD) and exported at 1 Hz frequency from the onset of each protocol after warm-up had been completed. Exercise protocols were: 30 min of moderate intensity (65% VO_2peak_) steady state exercise (MICT); four 4 min high intensity (85% HR_max_) interval bouts (HIIT) separated by 3 min active recovery at 50 W and four 30 s supramaximal (200% W_max_) sprint intervals (SIT) separated by 4.5 min active recovery at 50 W, with all protocols followed by 3 min of active recovery at 50 W. Data are presented as average ± 95% confidence intervals (*n* = 18), across the duration of each protocol including active recovery periods, with resting values averaged over 30 s of seated rest presented for comparison. These time periods were identical to those used in calculation of area under the curve (AUC). Continuous effort during MICT and the timing of the four interval periods in HIIT and SIT are highlighted in pink.

Area under the curve analysis of blood displacement across all sessions was carried out in eighteen participants; four participants were not included in this analysis due to temporary signal drop out during one or more sessions, making the analysis of area under the curve across the full session unreliable. Displacement over the course of MICT was significantly higher than both HIIT (1.66 × 10^5^ ± 3.12 × 10^4^ cm vs. 1.36 × 10^5^ ± 2.23 × 10^4^ cm, *p* < 0.001) and SIT (8.14 × 10^4^ ± 1.46 × 10^4^ cm, *p* < 0.001) exercise protocols. Figure 4 shows the mean MCAv across the period used in AUC analysis, in comparison to resting values, demonstrating the difference in average response between the three exercise protocols and the unique pattern of response between sprint bouts and immediate recovery period.

Heart rate data showed a clear pattern that was dependent on the intensity of the exercise stimulus within each of the protocols. Significant increases were seen from rest to the end of the warm up across all exercise protocols (all *p* < 0.001, Table 2), whilst the elevation in heart rate was significantly higher during the warm up in SIT than in MICT (126 ± 15 vs. 118 ± 12 beat⋅min^–1^, *p* = 0.003), but not in comparison to HIIT (121 ± 12 beat⋅min^–1^, *p* = 0.057). All three protocols saw a significant increase in HR from warm up to exercise (all *p* < 0.001), with significantly lower HR in MICT across all time points than both HIIT (minimum difference of Δ16 ± 12 beats⋅min^–1^, *p* < 0.001) and SIT (minimum Δ21 ± 14 beat⋅min^–1^, *p* < 0.001). Within each protocol, no change was seen across time points in MICT or SIT, however, HIIT bout 1 exhibited significantly lower HR (159 ± 10 beats⋅min^–1^) than in the subsequent bouts (bout 2: 167 ± 10 beats⋅min^–1^; bout 3: 170 ± 12 beats⋅min^–1^; bout 4: 172 ± 11 beats⋅min^–1^, all *p* < 0.04 from bout 1).

### Protocol-Dependent Changes in Circulating Factors

Blood samples were drawn from all 24 participants across all time points, with the exception of the final time point in MICT for one participant (cannula blockage). Of these, samples fell outside the limit of detection for the given test in three participants for BDNF (*n* = 21), seven participants for IGF-1 (*n* = 17), nine participants for VEGF (*n* = 15), and none for lactate (*n* = 24). Sample loss post collection for one participant for the SIT protocol resulted in a reduction in sample number for the analysis of VEGF (SIT: *n* = 14) and blood lactate (SIT: *n* = 23). No significant differences were seen in baseline measures for any of the blood markers (*p* > 0.05, Table 1). Model validation resulted in the inclusion of sex as a fixed factor in linear mixed modeling for BDNF alone, whilst analyses of data for IGF-1, VEGF, and blood lactate were carried out using base models alone.

**FIGURE 5:**
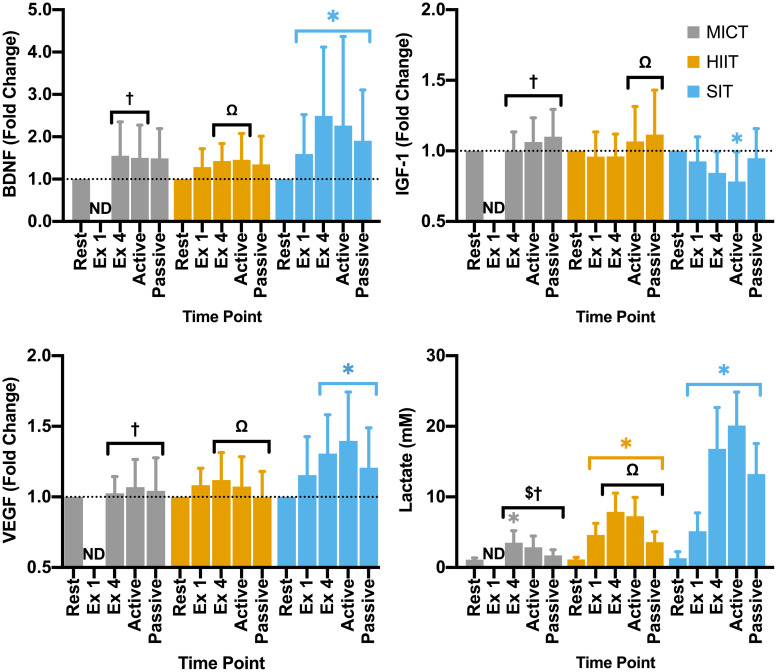
Blood plasma samples were taken during seated rest (Rest); immediately after the first interval exercise bout (Ex 1; HIIT and SIT only); at the end of exercise (Ex 4, end of active recovery (Active); and the end of passive recovery (Passive). Brain-derived neurotrophic factor (BDNF; *n* = 21), insulin-like growth factor 1 (IGF-1; *n* = 17), and vascular endothelial growth factor [VEGF; *n* = 15 (14 in SIT)] were analyzed by ELISA. Blood lactate [*n* = 24 (23 in SIT)] was analyzed using automated photometric analysis. Data are presented as mean ± *SD*. Significance (analyzed by linear mixed model, *p* < 0.05) between resting values and subsequent time points is denoted by *. Significant differences between protocols are denoted by ^$^ between MICT and HIIT; ^†^ between MICT and SIT; and ^Ω^ between HIIT and SIT. ND, not determined.

**FIGURE 6:**
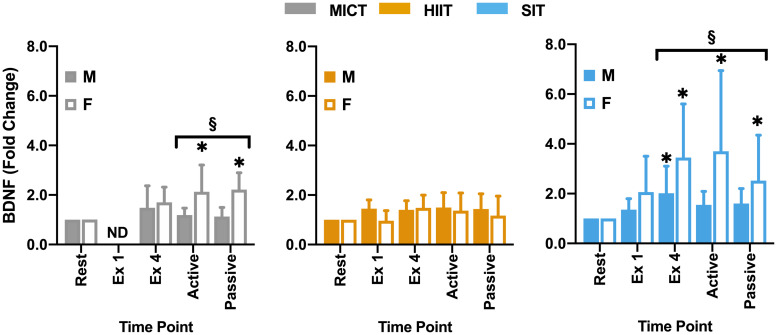
Blood plasma samples were taken were taken during seated rest (Rest); immediately after the first interval exercise bout (Ex 1; HIIT and SIT only); at the end of exercise (Ex 4); end of active recovery (Active)’ and end of passive recovery (Passive). Brain-derived neurotrophic factor (BDNF; *n* = 21, 7 female) was analyzed by ELISA. Data are presented as mean ± *SD*. Significance (analyzed by linear mixed model, *p* < 0.05) between resting values and subsequent time points is denoted by *. Significant differences between sexes at a given time point are denoted by ^§^. M, Male; F, Female; ND, not determined.

Within the full cohort, significant changes in BDNF were seen in SIT alone, with a significant elevation from rest at all later time points, peaking at the end of exercise (Δ149 ± 162% from rest, Figure 5) and remaining significantly elevated through to the end of passive recovery (all *p* ≤ 0.040). While baseline measures of BDNF did not differ significantly between male and female participants, significant differences were seen between sexes in both MICT and SIT exercise protocols (Figure 6). Females exhibited significantly greater increases in BDNF compared to males during active (*p* = 0.021) and passive recovery (*p* < 0.001) in MICT and from the end of exercise onwards in SIT (*p* < 0.001). Marked differences in the response to each protocol were also seen between sexes. Within the female subgroup, increases in BDNF from rest were seen following active recovery (*p* = 0.052), reaching significance at the end of passive recovery (*p* = 0.028) during MICT; significant elevations were also seen from the end of exercise onwards, peaking at active recovery (*p* ≤ 0.002) during SIT. Whereas within the male cohort, changes in BDNF levels were only significant between rest and end of exercise in the SIT protocol (*p* = 0.005).

Changes in IGF-1 across the duration of visits were only found to be significant in the SIT protocol, with a pattern of reduction across the exercise period reaching significance at the end of active recovery (*p* = 0.002, Figure 5). While not significantly elevated from baseline values, changes in IGF-1 for both MICT and HIIT were relatively higher than SIT at the end of active (*p* < 0.001) and passive recovery (MICT vs. SIT; *p* = 0.023 and HIIT vs. SIT; *p* = 0.011), and at the end of exercise for MICT compared to SIT (*p* = 0.018).

Significant changes in VEGF from baseline were only observed for the SIT protocol, with significant elevations in VEGF seen from the end of exercise onwards (>23%; *p* ≤ 0.015, Figure 5). Specifically, a significant difference in VEGF was seen between the immediate impact of a single SIT bout and further elevations in VEGF at the end of exercise (*p* = 0.017) and active recovery (*p* < 0.001). At all three of these time points, fold-change in VEGF was significantly greater than that of either MICT (*p* ≥ 0.036) or HIIT (*p* ≥ 0.026).

All three protocols increased blood lactate levels, but the magnitude of these changes varied between protocols. Specifically, while a significant elevation was seen in MICT at the end of exercise alone (*p* = 0.005), changes in HIIT and SIT were significant at all time points during and following the exercise stimulus (Figure 5). Further, whilst similar increases in lactate from rest were seen following the first bout of both HIIT (Δ3.47 ± 1.47 mM, *p* < 0.001) and SIT (Δ3.84 ± 2.97 mM, *p* < 0.001), significant divergence between these exercise protocols was seen after this time point. Elevations in blood lactate after the final bout of SIT were more than 2-fold greater than those seen in HIIT (Δ15.51 ± 6.14 vs. Δ6.95 ± 2.35 mM, *p* < 0.001), with lactate peaking from rest in SIT at the end of active recovery (Δ18.81 ± 4.91 mM, *p* < 0.001) before declining during passive recovery in both the HIIT and SIT protocols (Figure 5).

## Discussion

The primary purpose of this study was to compare how differences in exercise strategy may alter acute cerebrovascular responses and impact key circulating signaling factors with neurotrophic potential. Our main findings show that: (a) significant elevations in MCAv during MICT were matched by similar elevations in cerebral blood velocity for each bout of HIIT; (b) SIT elicited a unique response in MCAv, with only the initial bout inducing significant elevations in blood velocity from rest and a rebound in MCAv occurring immediately following each SIT bout; and (c) SIT alone drove significant changes in circulating levels of all investigated markers (BDNF, IGF-1, VEGF, and blood lactate). These data demonstrate that both the cerebrovascular and circulating signal factor responses to exercise are highly dependent on the protocol that is being undertaken. As a result, it is essential that we consider the relative contribution that hemodynamic and circulating factor responses have on the brain and its vasculature to fully understand the efficacy of different exercise strategies.

### Hemodynamic Responses to Acute Exercise Protocols

Our findings demonstrate that both MICT and HIIT exercise protocols elicited a similar magnitude of increase in cerebral blood velocity, although the pattern, duration, and total blood displacement over these two protocols varied (as demonstrated in Figure 4). The importance of the total shear stimulus in comparison to peak changes has been demonstrated over short time periods (up to 60 s) in brachial artery flow mediated dilatory (FMD) responses (Pyke and Tschakovsky, 2007), but to the best of our knowledge has not been investigated over longer time periods, such as that represented here. While Hoiland et al. (2017) demonstrated the capacity for transient periods of hypercapnia (30 s) to induce an FMD response within the cerebrovasculature, a recent comparison of brachial and internal carotid artery FMD showed that the magnitude and pattern of dilatory responses was distinctly different between the two vessels (Carr et al., 2020) and as such the impact of peak and total shear stimulus within the cerebrovasculature may be distinct from that seen within the periphery. Therefore, whether exercise-induced observations, both acute and chronic, based on FMD responses in the periphery are translated to FMD responses in the cerebrovasculature remain to be determined. This distinction is particularly pertinent in relation to the SIT protocol, as total blood displacement was the lowest overall (as reflected by AUC), yet changes over the first sprint interval and immediate post-bout were among the largest changes at any time point across all three protocols (see Figure 4).

The similarity in the acute responses between MICT and HIIT within this study have clear clinical ramifications, as these data indicate that an interval-based approach has the potential to elicit comparable beneficial changes in the cerebrovasculature. Additionally, the slight differences seen in the initial bout of HIIT are likely linked to the lower heart rate within this bout, resulting from an underestimation of the required exercise intensity to elicit 85% HR_max_, suggesting that a greater response may be seen at a lower intensity. This adds to a growing body of evidence supporting the utilization of interval-based exercise to target both general and vascular health outcomes (Weston et al., 2014; Pedersen and Saltin, 2015), and complements the recent findings of Klein et al. (2019) who demonstrated comparable responses to 10 min of work-matched continuous compared to intermittent exercise. This is an important step in increasing our understanding of how differences in the exercise protocol may affect cerebrovascular function (Calverley et al., 2020). Future work is essential to understand the impact that acute differences in exercise protocol response may have on changes in cerebrovascular function over a training period, particularly given the potential for acute responses to act as markers of possible adaptive benefits (Dawson et al., 2018; Voss et al., 2020).

As expected, suppression of exercise-induced increases in MCAv across the SIT protocol matched the overall lowering of P_ET_CO_2_, consistent with the hyperventilatory hypocapnia associated with high intensity exercise (Ogoh and Ainslie, 2010) and the central role of changes in arterial CO_2_ in regulating cerebral blood flow (reviewed in Smith and Ainslie, 2017). Interestingly, the rebound in MCAv immediately following each SIT bout also matched changes in P_ET_CO_2_ (Figure 3), while the greatest increases in both MCAv and P_ET_CO_2_ were observed within the first bout. Thus, MCAv responses during each bout and across all bouts appear to be strongly influenced by the changes in P_ET_CO_2_. While not investigated within the current study, oxidative-nitrosative stress has previously been linked to reductions in brachial artery endothelium-dependent vasodilation (Goto et al., 2003) and impaired dynamic cerebral autoregulation (Bailey et al., 2011) following high intensity exercise, and as such may also play some role in the MCAv response we observed with the SIT protocol. However, further research is needed to determine the oxidative-nitrosative stress response to interval-based high intensity exercise and the role that this plays within the cerebrovasculature.

Labrecque et al. (2020) previously demonstrated a similar rebound response with a single 30 s sprint bout and suggested that delays in the cerebral autoregulation may also contribute to this MCAv rebound, which, given the large elevations in blood pressure with SIT exercise, seems likely. Unfortunately, we were not able to accurately determine blood pressure during sprint intervals at the intensity utilized within the current study. Interestingly, rebound responses were not observed with the longer intervals used in the HIIT protocol. Therefore, future research is needed to fully understand the potential for differences in both intensity and duration of the interval to maximize the potential benefits of sprint-interval exercise, including with reliable measuring of blood pressure during such sprint efforts.

### Sprint-Based Intervals Elicit Unique Patterns in Neurotrophic and Signaling Factor Response

Contrasting the changes seen in MCAv, SIT alone induced significant changes in all assessed neurotrophic factors. Given the potential interaction between changes in these factors and exercise-induced improvements in cognitive function (Lista and Sorrentino, 2010; Skriver et al., 2014), these findings raise the possibility that exercise protocol choice may alter the profile of exercise-related benefits in brain health. Whilst a clear response was seen in blood lactate across all protocols, those seen in SIT were greater than in either the MICT or HIIT exercise protocols, and this may have widespread benefits as lactate serves as a signaling molecule (Nalbandian and Takeda, 2016; Morland et al., 2017). Additionally, elevations in lactate have been shown to drive subsequent elevations in circulating VEGF (Sameer Kumar et al., 2007) and BDNF (Schiffer et al., 2011), and as such may be key to changes in VEGF and BDNF being seen primarily within the SIT protocol alone.

The potential for lactate to stimulate VEGF release, alongside intensity-dependent upregulation within the skeletal muscle (Hoier and Hellsten, 2014), may help to explain the significant elevations in SIT alone. The lack of VEGF modulation by either MICT or HIIT adds to previous literature showing contrasting findings across a range of exercise protocols and cohorts, likely due to the influence of confounding factors (Kraus et al., 2004; Skriver et al., 2014; Basso and Suzuki, 2017). While it was beyond the scope of this study to determine the influence of all confounding factors, both sex and fitness were taken into consideration for model fitting and neither resulted in improved model fit. Based on previous research, low basal levels of VEGF in combination with high interindividual variability may explain why a number of samples were found to fall below the limit of detection during analysis (Jelkmann, 2001). Despite this reduction in sample size, significant increases were seen with SIT, indicating that SIT may induce a more consistent response in VEGF compared to lower intensity exercise. These findings add to a growing body of evidence that high intensity, interval-based conditions can have a significantly greater impact on transient responses in circulating VEGF (Wahl et al., 2011; Kujach et al., 2020).

Significant decreases in IGF-1 during SIT, alongside a pattern of suppression in HIIT, add to inconsistent responses in IGF-1 seen in previous research in which studies have demonstrated both a lack of response (Wahl et al., 2010) and significant elevation in IGF-1 with sprint-based exercise (Kujach et al., 2020). Interestingly, within the current study, IGF-1 does appear to be rising during the recovery period of both interval-based protocols. Previous research has demonstrated dichotomous results in measures of IGF-1 taken during recovery, with those showing increases during recovery finding peak changes within an hour of exercise (Kraemer et al., 2017). The relationship between IGF-1 and its binding proteins in regulating signaling (Kraemer et al., 2017); alongside the capacity for peripheral IGF-1 to be regulated by and transported into the brain in animal models (Fernandez and Torres-Alemán, 2012; Vecchio et al., 2018) adds further complexity to exercise associated responses. For this reason, it is essential that future studies explore the role that exercise protocol choices play in modulating IGF-1 and associated binding proteins, particularly over longer periods of recovery.

Responses in BDNF within the current study are consistent with previous research in relation to the response to SIT (Kujach et al., 2020) and the relative lack of response at lower intensities (Nofuji et al., 2012; Reycraft et al., 2020). One possible explanation for this lack of response at lower intensities is the relatively short half-life of BDNF in the circulation of ∼2.7 min reported by some authors (Pardridge et al., 1994), which would necessitate very high levels of release in order for accumulation of BDNF to be detected in plasma. However, more recent studies have disputed this half-life time, demonstrating half-lives as long as 60 min (Pan et al., 1998; Khalin et al., 2016). In addition, the potential for lactate to upregulate BDNF in a similar manner to VEGF (Schiffer et al., 2011) also raises the potential role that intensity-related elevations in lactate may play in BDNF elevations. Interestingly, a significant difference was seen between male and female participants within the current study, with female participants showing greater responses in the recovery period during SIT and significant elevations in MICT that were not seen in male participants or the cohort as a whole (Figure 6). Previous studies have highlighted the potential for sex differences both at rest (Lommatzsch et al., 2005; Piccinni et al., 2008) and during exercise (Serra-Millàs, 2016; Dinoff et al., 2017). While in a recent meta-analysis, Dinoff et al. (2017) found significant elevations in BDNF in males alone, the authors highlighted that this may be due in part to a relative lack of studies including female cohorts. In combination with the contrasting findings of the present study and a general lack of studies across different exercise protocols, there is a clear need for further research into the potential role of sex differences in exercise-related BDNF responses. Within the current study female participation was not restricted by oral contraceptive use or menstrual cycle phases, so the role that cycle phase plays in these differences cannot be fully understood. This is particularly pertinent given the significant variations across the menstrual cycle highlighted by Begliuomini et al. (2007), thus further research is needed to explore the influence that this basal variability may play in exercise-induced responses within females and in comparison to male cohorts. The lack of BDNF modulation by HIIT also raises interesting questions for future study as to the role of exercise duration and intensity in stimulating changes in BDNF, with consideration as to how plasma half-life may dictate the capacity for exercise to drive accumulation within circulation.

### Limitations and Future Directions

While growing evidence supports the utilization of interval-based exercise, it is important to consider the potential risks of these strategies against the physiological benefits they may provide (Lucas et al., 2015; Labrecque et al., 2020). Encouragingly, recent studies into the risk between interval-based and traditional continuous exercise protocols for cardiovascular disease cohorts have shown that the two are of similar risk (Rognmo et al., 2012; Wewege et al., 2018). Our findings, alongside the limited risk that clinical guideline-based intervals appear to pose, do suggest that interval-based exercise can be safely used to positively impact cerebrovascular health. However, it is still important that the safety of such interventions are considered before they are applied across a broader population, including at-risk cohorts.

The utilization of TCD to measure blood velocity as a proxy of cerebral blood flow has been widely debated (Ainslie and Hoiland, 2014). The potential for the MCA to change in diameter during exercise has significant ramifications for the use of TCD, particularly as the nature of the diameter change may depend on the specific exercise stimulus and these concerns must be taken into consideration when utilizing TCD (Hoiland and Ainslie, 2016; Verbree et al., 2017). While alternative methods such as MRI, fNIRS, and duplex ultrasound may present a more accurate or in depth examination of the cerebrovasculature, at present these methods cannot be employed across the range of exercise protocols examined in the current study (Ainslie and Hoiland, 2014).

The potential influence of changes in hemoconcentration as a result of fluid shift during exercise was not considered within the current study. While previous research has shown greater levels of hemoconcentration at higher intensities (Wahl et al., 2010; Bloomer and Farney, 2013), the lack of responses in either MICT or HIIT, alongside significant reductions in IGF-1, contradict the potential for circulating responses to be purely hemoconcentration-dependent. Additionally, measures of BDNF were taken in plasma samples alone and not in serum, which the latter previously has been shown to generate significantly higher yields of BDNF due to the contribution of platelet-derived BDNF (Serra-Millàs, 2016). However, our aim within the current study was to identify responses within the circulating levels of neurotrophic factors and their release from tissue and as such plasma-based measures of BDNF were chosen. While care was taken in the preparation of plasma samples, so as to avoid the inclusion of platelets within the final sample, we did not include a second centrifugation step to eliminate any residual platelets from samples and as such this may have affected BDNF and other measures sensitive to platelet activity. However, preparation of plasma was carried out at higher speeds in a similar manner to a recently validated protocol for the removal of platelets by single-step centrifugation (Rikkert et al., 2020), although this protocol utilized a 20-minute centrifugation, compared to the 10-minute period used within the current study.

As already discussed, female participants were included regardless of oral contraceptive usage and menstrual phase. While this limits our ability to determine the influence of sex hormones or menstrual cycle, this decision was made to address the studies aims in including free-living individuals and to maximize recruitment of female participants (Stanhewicz and Wong, 2020). Furthermore, while numerous strategies have been suggested to control for the menstrual cycle within studies, the variability between individuals makes many of these strategies unreliable (Häggström, 2014; Sims and Heather, 2018). Nevertheless, our findings demonstrate the potential for sex differences to influence acute exercise responses within the vasculature, warranting further exploration of this interaction.

While this study demonstrates the clear difference in acute responses, it is not yet clear whether the differences between these acute bouts result in significant differences in a chronic context. As such, although growing evidence supports the concept that acute responses are often indicative of the chronic impact of interventions (Dawson et al., 2018; Voss et al., 2020), the exploration of the response to a prolonged training period across all three exercise approaches is clearly warranted. Similarly, the population studied within the current work were healthy, young, and active, and so future work is required to identify how differences across the population may alter this response.

## Conclusion

In conclusion, similar acute hemodynamic responses are seen between traditional continuous and more recent guideline-based interval exercise strategies (4 × 4 min bouts at 85% HR_max_), indicating that this type of interval-based exercise approach may also be capable of inducing similar changes in cerebrovascular function over chronic training periods. While sprint-based intervals drove relatively limited changes in blood velocity compared to guideline-based interval and traditional continuous exercise strategies, these intervals induced large changes in circulating factors with a potential to significantly impact cerebrovascular health and function. This dichotomy in response between exercise strategies may provide an alternate or adjunct means for improving brain health through combining approaches to physical activity within interventions.

## Data Availability Statement

The raw data supporting the conclusions of this article will be made available by the authors, without undue reservation.

## Ethics Statement

The studies involving human participants were reviewed and approved by the University of Birmingham Ethics Committee. The participants provided their written informed consent to participate in this study.

## Author Contributions

Experiments were carried out in the School of Sport, Exercise and Rehabilitation Sciences at the University of Birmingham, UK. SW, RF, and SL: conception and design of the experiments. SW, BS, RF, RL, SL, NC, CR, and HM: pilot testing, design revision, and experiment preparation. SW, BS, RF, RL, and SL: data collection. SW and SL: data processing and analysis. SW, HM, and SL: manuscript preparation. All co-authors read, contributed with comments, and approved the final version of the manuscript.

## Conflict of Interest

The authors declare that the research was conducted in the absence of any commercial or financial relationships that could be construed as a potential conflict of interest.

## References

[B1] Ainslie P. N. Hoiland R. L. (2014). Transcranial doppler ultrasound: valid, invalid, or both? *J. Appl. Physiol.* 117 1081–1083. 10.1152/japplphysiol.00854.2014 25257879

[B2] Alfini A. J. Weiss L. R. Nielson K. A. Verber M. D. Smith J. C. (2019). Resting cerebral blood flow after exercise training in mild cognitive impairment alfonso. *J. Alzheimers Dis.* 67 671–684. 10.3233/jad-180728 30636734 PMC6444938

[B3] Bailey D. M. Evans K. A. Mceneny J. Young I. S. Hullin D. A. James P. E. (2011). Exercise-induced oxidative-nitrosative stress is associated with impaired dynamic cerebral autoregulation and blood-brain barrier leakage. *Exp. Physiol.* 96 1196–1207. 10.1113/expphysiol.2011.060178 21841038

[B4] Bailey D. M. Marley C. J. Brugniaux J. V. Hodson D. New K. J. Ogoh S. (2013). Elevated aerobic fitness sustained throughout the adult lifespan is associated with improved cerebral hemodynamics. *Stroke* 44 3235–3238. 10.1161/strokeaha.113.002589 23963329

[B5] Basso J. C. Suzuki W. A. (2017). The effects of acute exercise on mood, cognition, neurophysiology, and neurochemical pathways: a review. *Brain Plast* 2 127–152. 10.3233/bpl-160040 29765853 PMC5928534

[B6] Begliuomini S. Casarosa E. Pluchino N. Lenzi E. Centofanti M. Freschi L. (2007). Influence of endogenous and exogenous sex hormones on plasma brain-derived neurotrophic factor. *Hum. Reprod.* 22 995–1002. 10.1093/humrep/del479 17251358

[B7] Bloomer R. J. Farney T. M. (2013). Acute plasma volume change with high-intensity sprint exercise. *J. Strength Cond. Res.* 27 2874–2878. 10.1519/jsc.0b013e318282d416 23302756

[B8] Borg G. A. V. (1982). Psychophysical bases of perceived exertion. *Med. Sci. Sports Exerc.* 14 377–381. 7154893

[B9] Brassard P. Tymko M. M. Ainslie P. N. (2017). Sympathetic control of the brain circulation: appreciating the complexities to better understand the controversy. *Auton. Neurosci. Basic. Clin.* 207 37–47. 10.1016/j.autneu.2017.05.003 28506501

[B10] Burley C. V. Bailey D. M. Marley C. J. Lucas S. J. E. (2016). Brain train to combat brain drain; focus on exercise strategies that optimize neuroprotection. *Exp. Physiol.* 101 1178–1184. 10.1113/ep085672 27443587

[B11] Cai H. Li G. Hua S. Liu Y. Chen L. (2017). Effect of exercise on cognitive function in chronic disease patients: a meta-analysis and systematic review of randomized controlled trials. *Clin. Interv. Aging* 2017 773–783. 10.2147/cia.s135700 28546744 PMC5436795

[B12] Calverley T. A. Ogoh S. Marley C. J. Steggall M. Marchi N. Brassard P. (2020). HIITing the brain with exercise: mechanisms, consequences and practical recommendations. *J. Physiol.* 598 2513–2530. 10.1113/jp275021 32347544

[B13] Carr J. M. J. R. Hoiland R. L. Caldwell H. G. Coombs G. B. Howe C. A. Tremblay J. C. (2020). Internal carotid and brachial artery shear-dependent vasodilator function in young healthy humans. *J. Physiol.* 0 1–18. 10.1007/978-3-319-70267-4_129-1 32901919

[B14] Carro E. Nuñez A. Busiguina S. Torres-Aleman I. (2000). Circulating insulin-like growth factor I mediates effects of exercise on the brain. *J. Neurosci.* 20 2926–2933. 10.1523/jneurosci.20-08-02926.2000 10751445 PMC6772191

[B15] Costello E. Kafchinski M. Vrazel J. Sullivan P. (2011). Motivators, barriers, and beliefs regarding physical activity in an older adult population. *J. Geriatr. Phys. Ther.* 34 138–147. 10.1519/jpt.0b013e31820e0e71 21937904

[B16] Curtelin D. Morales-Alamo D. Torres-Peralta R. Rasmussen P. Martin-Rincon M. Perez-Valera M. (2018). Cerebral blood flow, frontal lobe oxygenation and intra-arterial blood pressure during sprint exercise in normoxia and severe acute hypoxia in humans. *J. Cereb. Blood Flow Metab.* 38 136–150. 10.1177/0271678x17691986 28186430 PMC5757439

[B17] Dawson E. A. Cable N. T. Green D. J. Thijssen D. H. J. (2018). Do acute effects of exercise on vascular function predict adaptation to training? *Eur. J. Appl. Physiol.* 118 523–530. 10.1007/s00421-017-3724-8 29234916 PMC5805792

[B18] Dinoff A. Herrmann N. Swardfager W. Lanctôt K. L. (2017). The effect of acute exercise on blood concentrations of brain-derived neurotrophic factor in healthy adults: a meta-analysis. *Eur. J. Neurosci.* 46 1635–1646. 10.1111/ejn.13603 28493624

[B19] Fernandez A. M. Torres-Alemán I. (2012). The many faces of insulin-like peptide signalling in the brain. *Nat. Rev. Neurosci.* 13 225–239. 10.1038/nrn3209 22430016

[B20] Garber C. E. Blissmer B. Franklin B. Nieman D. C. (2011). Quantity and quality of exercise for developing and maintaining cardiorespiratory, neuromotor fitness in apparently healthy adults: guidance for prescribing exercise. *Med. Sci. Sport Exerc.* 43 1334–1359. 10.1249/mss.0b013e318213fefb 21694556

[B21] Geiseler S. J. Morland C. (2018). The janus face of VEGF in stroke. *Int. J. Mol. Sci.* 19 1–20. 10.3390/ijms19051362 PMC5983623 29734653

[B22] Gibala M. J. Little J. P. (2020). Physiological basis of brief vigorous exercise to improve health. *J. Physiol.* 598 61–69. 10.1113/jp276849 31691289

[B23] Góra-Kupilas K. Jośko J. (2005). The neuroprotective function of vascular endothelial growth factor (VEGF). *Folia Neuropathol.* 43 31–39. 15827888

[B24] Goto C. Higashi Y. Kimura M. Noma K. Hara K. Nakagawa K. (2003). Effect of different intensities of exercise on endothelium-dependent vasodilation in humans. *Circulation* 108 530–535. 10.1161/01.cir.0000080893.55729.28 12874192

[B25] Green D. J. Hopman M. T. E. Padilla J. Laughlin M. H. Thijssen D. H. J. (2017). Vascular adaptation to exercise in humans: role of hemodynamic stimuli. *Physiol. Rev.* 97 495–528. 10.1152/physrev.00014.2016 28151424 PMC5539408

[B26] Häggström M. (2014). Reference ranges for estradiol, progesterone, luteinizing hormone and follicle-stimulating hormone during the menstrual cycle. *Wiki J. Med.* 1 1–5.

[B27] Hoier B. Hellsten Y. (2014). Exercise-induced capillary growth in human skeletal muscle and the dynamics of VEGF. *Microcirculation* 21 301–314. 10.1111/micc.12117 24450403

[B28] Hoiland R. L. Ainslie P. N. (2016). The middle cerebral artery diameter does change during alterations in arterial blood gases and blood pressure. *J. Physiol.* 594 4073–4075. 10.1113/jp271981 27010010 PMC4806217

[B29] Hoiland R. L. Smith K. J. Carter H. H. Lewis N. C. S. Tymko M. M. Wildfong K. W. (2017). Shear-mediated dilation of the internal carotid artery occurs independent of hypercapnia. *Am. J. Physiol. Hear Circ. Physiol.* 313 H24–H31. 10.1152/ajpheart.00119.2017 28389602

[B30] Hughson R. L. Weisiger K. H. Swanson G. D. (1987). Blood lactate concentration increases as a continuous function in progressive exercise. *J. Appl. Physiol.* 62 1975–1981. 10.1152/jappl.1987.62.5.1975 3597269

[B31] Jelkmann W. (2001). Pitfalls in the measurement of circulating vascular endothelial growth factor. *Clin. Chem.* 47 617–623. 10.1093/clinchem/47.4.617 11274009

[B32] Jeon Y. K. Ha C. H. (2017). The effect of exercise intensity on brain derived neurotrophic factor and memory in adolescents. *Environ. Health Prev. Med.* 22 1–6. 29165142 10.1186/s12199-017-0643-6 PMC5664787

[B33] Jin K. Zhu Y. Sun Y. Mao X. O. Xie L. Greenberg D. A. (2002). Vascular endothelial growth factor (VEGF) stimulates neurogenesis in vitro and in vivo. *Proc. Natl. Acad. Sci. U.S.A.* 99 11946–11950. 10.1073/pnas.182296499 12181492 PMC129374

[B34] Khalin I. Alyautdin R. Wong T. W. Gnanou J. Kocherga G. Kreuter J. (2016). Brain-derived neurotrophic factor delivered to the brain using poly (lactide-co-glycolide) nanoparticles improves neurological and cognitive outcome in mice with traumatic brain injury. *Drug Deliv.* 23 3520–3528. 10.1080/10717544.2016.1199609 27278330

[B35] Klein A. B. Williamson R. Santini M. A. Clemmensen C. Ettrup A. Rios M. (2011). Blood BDNF concentrations reflect brain-tissue BDNF levels across species. *Int. J. Neuropsychopharmacol.* 14 347–353. 10.1017/s1461145710000738 20604989

[B36] Klein T. Bailey T. G. Abeln V. Schneider S. Askew C. D. (2019). Cerebral blood flow during interval and continuous exercise in young and old men. *Med. Sci. Sports Exerc.* 51 1523–1531. 10.1249/mss.0000000000001924 30768552

[B37] Kraemer W. J. Ratamess N. A. Nindl B. C. (2017). Recovery responses of testosterone, growth hormone, and IGF-1 after resistance exercise. *J. Appl. Physiol.* 122 549–558. 10.1152/japplphysiol.00599.2016 27856715

[B38] Kramer A. F. Erickson K. I. Colcombe S. J. (2006). Exercise, cognition, and the aging brain. *J. Appl. Physiol.* 101 1237–1242. 16778001 10.1152/japplphysiol.00500.2006

[B39] Kraus R. M. Stallings H. W. Yeager R. C. Gavin T. P. (2004). Circulating plasma VEGF response to exercise in sedentary and endurance-trained men. *J. Appl. Physiol.* 96 1445–1450. 10.1152/japplphysiol.01031.2003 14660505

[B40] Kraus W. E. Powell K. E. Haskell W. L. Janz K. F. Campbell W. W. Jakicic J. M. (2019). Physical activity, all-cause and cardiovascular mortality, and cardiovascular disease. *Med. Sci. Sports Exerc.* 51 1270–1281. 31095084 10.1249/MSS.0000000000001939 PMC6527136

[B41] Kujach S. Olek R. A. Byun K. Suwabe K. Sitek E. J. Ziemann E. (2020). Acute sprint interval exercise increases both cognitive functions and peripheral neurotrophic factors in humans: the possible involvement of lactate. *Front. Neurosci.* 13:1455. 10.3389/fnins.2019.01455 32038149 PMC6989590

[B42] Labrecque L. Drapeau A. Rahimaly K. Imhoff S. Billaut F. Brassard P. (2020). Comparable blood velocity changes in middle and posterior cerebral arteries during and following acute high-intensity exercise in young fit women. *Physiol. Rep.* 8 1–10. 10.1096/fasebj.2020.34.s1.08667 PMC7186567 32342622

[B43] Lauritzen K. H. Morland C. Puchades M. Holm-Hansen S. Hagelin E. M. Lauritzen F. (2014). Lactate receptor sites link neurotransmission, neurovascular coupling, and brain energy metabolism. *Cereb Cortex* 24 2784–2795. 10.1093/cercor/bht136 23696276

[B44] Lewitt M. S. Boyd G. W. (2019). The role of insulin-like growth factors and insulin-like growth factor–binding proteins in the nervous system. *Biochem. Insights* 12:117862641984217. 10.1177/1178626419842176 31024217 PMC6472167

[B45] Lista I. Sorrentino G. (2010). Biological mechanisms of physical activity in preventing cognitive decline. *Cell Mol. Neurobiol.* 30 493–503. 10.1007/s10571-009-9488-x 20041290 PMC11498799

[B46] Lommatzsch M. Zingler D. Schuhbaeck K. Schloetcke K. Zingler C. Schuff-Werner P. (2005). The impact of age, weight and gender on BDNF levels in human platelets and plasma. *Neurobiol. Aging* 26 115–123. 10.1016/j.neurobiolaging.2004.03.002 15585351

[B47] Lucas S. J. E. Cotter J. D. Brassard P. Bailey D. M. (2015). High-intensity interval exercise and cerebrovascular health: curiosity, cause, and consequence. *J. Cereb Blood Flow Metab.* 35 902–911. 10.1038/jcbfm.2015.49 25833341 PMC4640257

[B48] Morland C. Andersson K. A. Haugen O. P. Haugen A. Hadzic L. Gille A. (2017). Exercise induces cerebral VEGF and angiogenesis via the lactate receptor HCAR1. *Nat. Commun.* 8:15557. 10.1038/ncomms15557 PMC5457513 28534495

[B49] Nalbandian M. Takeda M. (2016). Lactate as a signaling molecule that regulates exercise-induced adaptations. *Biology (Basel)* 5 1–12. 10.3390/biology5040038 PMC5192418 27740597

[B50] Nofuji Y. Suwa M. Sasaki H. Ichimiya A. Nishichi R. Kumagai S. (2012). Different circulating brain-derived neurotrophic factor responses to acute exercise between physically active and sedentary subjects. *J. Sport Sci. Med.* 11 83–88. PMC3737858 24137066

[B51] O’Donovan G. Lee I. M. Hamer M. Stamatakis E. (2017). Association of “weekend warrior” and other leisure time physical activity patterns with risks for all-cause, cardiovascular disease, and cancer mortality. *JAMA Intern. Med.* 177 335–342. 10.1001/jamainternmed.2016.8014 28097313

[B52] Ogoh S. Ainslie P. N. (2010). Cerebral blood flow during exercise: mechanisms of regulation. *J. Appl. Physiol.* 1 1370–1380. 10.1152/japplphysiol.00573.2009 19729591

[B53] Ogoh S. Dalsgaard M. K. Yoshiga C. C. Dawson E. A. Keller D. M. Raven P. B. (2018). Dynamic cerebral autoregulation during exhaustive exercise in humans. *Am. J. Physiol. Heart Circ. Physiol.* 76107 1461–1467. 10.1152/ajpheart.00948.2004 15498819

[B54] Olsson A. K. Dimberg A. Kreuger J. Claesson-Welsh L. (2006). VEGF receptor signalling – In control of vascular function. *Nat. Rev. Mol. Cell Biol.* 7 359–371. 10.1038/nrm1911 16633338

[B55] Pan W. Banks W. A. Fasold M. B. Bluth J. Kastin A. J. (1998). Transport of brain-derived neurotrophic factor across the blood-brain barrier. *Neuropharmacology* 37 1553–1561. 10.1016/s0028-3908(98)00141-5 9886678

[B56] Pardridge W. M. Kang Y. S. Buciak J. L. (1994). Transport of human recombinant brain-derived neurotrophic factor (BDNF) through the rat blood-brain barrier in vivo using vector-mediated peptide drug delivery. *Pharm. Res.* 11 738–746. 8058646 10.1023/a:1018940732550

[B57] Pedersen B. K. Saltin B. (2015). Exercise as medicine – Evidence for prescribing exercise as therapy in 26 different chronic diseases. *Scand. J. Med. Sci. Sport* 25 1–72. 10.1111/sms.12581 26606383

[B58] Piccinni A. Marazziti D. Del Debbio A. Bianchi C. Roncaglia I. Mannari C. (2008). Diurnal variation of plasma brain-derived neurotrophic factor (BDNF) in humans: An analysis of sex differences. *Chronobiol. Int.* 25 819–826. 10.1080/07420520802387773 18780207

[B59] Pyke K. E. Tschakovsky M. E. (2007). Peak vs. total reactive hyperemia: Which determines the magnitude of flow-mediated dilation? *J. Appl. Physiol.* 102 1510–1519. 10.1152/japplphysiol.01024.2006 17170205

[B60] R Core Team (2019). *R: A Language and Environment for Statistical Computing.* Vienna: R Core Team.

[B61] Ramos J. S. Dalleck L. C. Tjonna A. E. Beetham K. S. Coombes J. S. (2015). The impact of high-intensity interval training versus moderate-intensity continuous training on vascular function: a systematic review and meta-analysis. *Sport Med.* 45 679–692. 10.1007/s40279-015-0321-z 25771785

[B62] Rasmussen P. Brassard P. Adser H. Pedersen M. V. Leick L. Hart E. (2009). Evidence for a release of brain-derived neurotrophic factor from the brain during exercise. *Exp. Physiol.* 94 1062–1069. 10.1113/expphysiol.2009.048512 19666694

[B63] Reycraft J. T. Islam H. Townsend L. K. Hayward G. C. Hazell T. J. Macpherson R. E. K. (2020). Exercise intensity and recovery on circulating brain-derived neurotrophic factor. *Med. Sci. Sports Exerc.* 52 1210–1217. 10.1249/mss.0000000000002242 31815833

[B64] Rikkert L. G. Coumans F. A. W. Hau C. M. Terstappen L. W. M. M. Nieuwland R. (2020). Platelet removal by single-step centrifugation. *Platelets* 17 1–4. 10.1080/09537104.2020.1779924 32552252

[B65] Rognmo Ø Moholdt T. Bakken H. Hole T. (2012). Cardiovascular risk of high- versus moderate-intensity aerobic exercise in coronary heart disease patients. *Circulation* 126 1436–1440. 10.1161/circulationaha.112.123117 22879367

[B66] Sameer Kumar V. B. Viji R. I. Kiran M. S. Sudhakaran P. R. (2007). Endothelial cell response to lactate: implucation of PAR modification of VEGF. *J. Cell Physiol.* 211 477–485. 10.1002/jcp.20955 17167776

[B67] Schiffer T. Schulte S. Sperlich B. Achtzehn S. Fricke H. Strüder H. K. (2011). Lactate infusion at rest increases BDNF blood concentration in humans. *Neurosci. Lett.* 488 234–237. 10.1016/j.neulet.2010.11.035 21094220

[B68] Serra-Millàs M. (2016). Are the changes in the peripheral brain-derived neurotrophic factor levels due to platelet activation? *World J. Psychiatry* 6:84. 10.5498/wjp.v6.i1.84 27014600 PMC4804271

[B69] Sims S. T. Heather A. K. (2018). Myths and methodologies: reducing scientific design ambiguity in studies comparing sexes and/or menstrual cycle phases. *Exp. Physiol.* 103 1309–1317. 10.1113/ep086797 30051938

[B70] Skriver K. Roig M. Lundbye-Jensen J. Pingel J. Helge J. W. Kiens B. (2014). Acute exercise improves motor memory: exploring potential biomarkers. *Neurobiol. Learn. Mem.* 116 46–58. 10.1016/j.nlm.2014.08.004 25128877

[B71] Smith K. J. Ainslie P. N. (2017). Regulation of cerebral blood flow and metabolism during exercise. *Exp. Physiol.* 102 1356–1371. 10.1113/ep086249 28786150

[B72] Stanhewicz A. E. Wong B. J. (2020). Counterpoint: Investigators should not control for menstrual cycle phase when performing studies of vascular control that include women. *J. Appl. Physiol.* 2:00427. 10.1152/japplphysiol.00427.2020 32702274

[B73] Stork M. J. Banfield L. E. Gibala M. J. Martin Ginis K. A. (2017). A scoping review of the psychological responses to interval exercise: is interval exercise a viable alternative to traditional exercise? *Health Psychol. Rev.* 11 324–344. 10.1080/17437199.2017.1326011 28460601

[B74] Symonds M. R. E. Moussalli A. (2011). A brief guide to model selection, multimodel inference and model averaging in behavioural ecology using Akaike’s information criterion. *Behav. Ecol. Sociobiol.* 65 13–21. 10.1007/s00265-010-1037-6

[B75] Tsai C. L. Wang C. H. Pan C. Y. Chen F. C. Huang T. H. Chou F. Y. (2014). Executive function and endocrinological responses to acute resistance exercise. *Front. Behav. Neurosci.* 8:262. 10.3389/fnbeh.2014.00262 25136300 PMC4117935

[B76] Vecchio L. M. Meng Y. Xhima K. Lipsman N. Hamani C. Aubert I. (2018). The neuroprotective effects of exercise: maintaining a healthy brain throughout aging. *Brain Plast* 4 17–52. 10.3233/bpl-180069 30564545 PMC6296262

[B77] Verbree J. Bronzwaer A. G. T. Van Buchem M. A. (2017). Middle cerebral artery diameter changes during rhythmic handgrip exercise in humans. *J. Cereb. Blood Flow Metab.* 37 2921–2927. 10.1177/0271678X16679419 27837189 PMC5536799

[B78] Voss M. W. Erickson K. I. Prakash R. S. Chaddock L. Kim J. S. Alves H. (2013a). Neurobiological markers of exercise-related brain plasticity in older adults. *Brain Behav. Immun.* 28 90–99. 10.1016/j.bbi.2012.10.021 23123199 PMC3544982

[B79] Voss M. W. Vivar C. Kramer A. F. van Praag H. (2013b). Bridging animal and human models of exercise-induced brain plasticity. *Trends Cogn. Sci.* 17 525–544. 10.1016/j.tics.2013.08.001 24029446 PMC4565723

[B80] Voss M. W. Weng T. B. Narayana-Kumanan K. Cole R. C. Wharff C. Reist L. (2020). Acute exercise effects predict training change in cognition and connectivity. *Med. Sci. Sports Exerc.* 52 131–140. 10.1249/mss.0000000000002115 31385912 PMC7753185

[B81] Wahl P. Jansen F. Achtzehn S. Schmitz T. Bloch W. Mester J. (2014). Effects of high intensity training and high volume training on endothelial microparticles and angiogenic growth factors. *PLoS One* 9:e96024. 10.1371/journal.pone.0096024 24770423 PMC4000202

[B82] Wahl P. Zinner C. Achtzehn S. Bloch W. Mester J. (2010). Effect of high- and low-intensity exercise and metabolic acidosis on levels of GH, IGF-I, IGFBP-3 and cortisol. *Growth Horm. IGF Res.* 20 380–385. 10.1016/j.ghir.2010.08.001 20801067

[B83] Wahl P. Zinner C. Achtzehn S. Mester J. (2011). Effects of acid – base balance and high or low intensity exercise on VEGF and bFGF. *Eur. J. Appl. Physiol.* 111 1405–1413. 10.1007/s00421-010-1767-1 21161264

[B84] Weston K. S. Wisløff U. Coombes J. S. (2014). High-intensity interval training in patients with lifestyle-induced cardiometabolic disease: a systematic review and meta-analysis. *Br. J. Sports Med.* 48 1227–1234. 10.1136/bjsports-2013-092576 24144531

[B85] Wewege M. A. Ahn D. Yu J. Liou K. Keech A. (2018). High-intensity interval training for patients with cardiovascular disease-is it safe? A systematic review. *J. Am. Heart Assoc.* 7 1–19. 10.1161/JAHA.118.009305 PMC6404189 30376749

[B86] Willie C. K. Colino F. L. Bailey D. M. Tzeng Y. C. Binsted G. Jones L. W. (2011). Utility of transcranial doppler ultrasound for the integrative assessment of cerebrovascular function. *J. Neurosci. Methods* 196 221–237. 10.1016/j.jneumeth.2011.01.011 21276818

