# Corrigendum: Cerebral Hemodynamic and Neurotrophic Factor Responses Are Dependent on the Type of Exercise

**DOI:** 10.3389/fphys.2021.659873

**Published:** 2021-02-24

**Authors:** Samuel R. Weaver, Bethany D. Skinner, Rhodri Furlong, Rebekah A. I. Lucas, N. Timothy Cable, Catarina Rendeiro, Helen M. McGettrick, Samuel J. E. Lucas

**Affiliations:** ^1^School of Sport, Exercise and Rehabilitation Sciences, College of Life and Environmental Sciences, University of Birmingham, Birmingham, United Kingdom; ^2^Centre for Human Brain Health, University of Birmingham, Birmingham, United Kingdom; ^3^College of Medical and Dental Sciences, Institute of Inflammation and Ageing, University of Birmingham, Birmingham, United Kingdom

**Keywords:** cerebrovascular, exercise, hemodynamic, neurotrophic factor, physiology

In the original article, there was a mistake in the legend for **Figure 3** as published. **Parts A and B were labeled in the incorrect order, the order of these panels within the figure was altered during reviewer responses and the figure legend was not updated correctly in the process**. The correct legend appears below.

**Figure 3. (A)** MCAv was determined continuously by transcranial doppler ultrasound (TCD) and presented as change in MCAv from rest in each protocol for each participant. (**B)** P_ET_CO_2_ was determined by measurement of breath-by-breath respiratory gas exchange and ventilation. Data were averaged for 1 min during seated rest (Rest); over 30 s during the final minute of four 4 min high intensity (85% HR_max_) interval bouts (HIIT; Ex 1 – 4); across the duration of four 30 s supramaximal (200% W_max_) sprint intervals (SIT; Ex 1 – 4), and over a 30 s period, 15 s into the recovery following each bout in both interval protocols (Reco 1 – 4). Data are presented as mean ± SD (*n* = 24). Significance (analyzed by linear mixed model, *p* < 0.05) between time points is denoted by ^*^ for differences from resting values;^€^ for differences between interval (ex) and recovery (reco). Significant differences between protocols are denoted by ^***Ω***^.

The authors apologize for this error and state that this does not change the scientific conclusions of the article in any way. The original article has been updated.

